# Supplementary material for: Using citizen science data to estimate trait and climate drivers of daily activity patterns in temperate butterflies
Source: PLoS One. 2025 Nov 21;20(11):e0335856. doi: 10.1371/journal.pone.0335856 (PMC12637895; doi:10.1371/journal.pone.0335856)
Supplement: S1 File — (DOCX) [file pone.0335856.s001.docx]

Supporting Information for “Using citizen science data to estimate trait and climate drivers of daily activity patterns in temperate butterflies” by Idec et al. (2025)


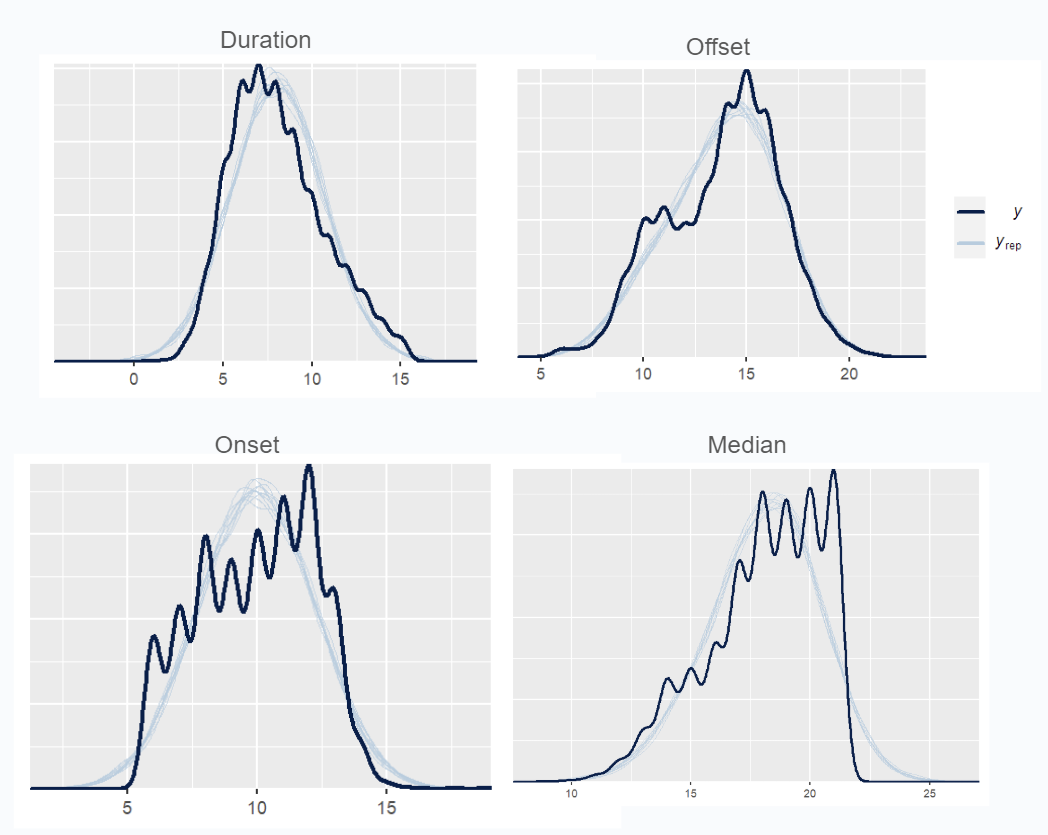


**SI Figure 1. Density-overlay posterior predictive checks for the top models for each response** using the pp_check() function in brms, assessing fit between data simulated using the model (light blue, y) and the real data (black line, yrep).


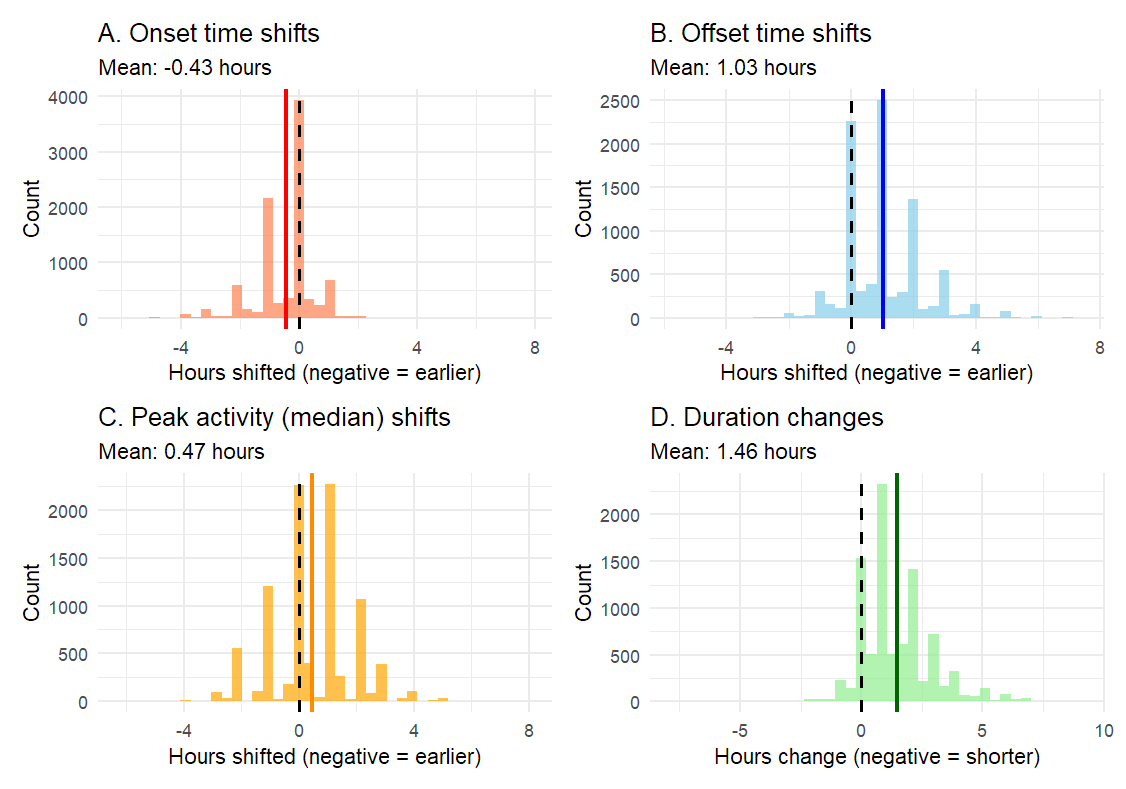


**SI Figure 2. Impact of observer bias correction on butterfly daily activity metrics.** Distribution of changes in activity timing and duration after applying bias correction to account for temporal sampling biases in iNaturalist observations. Each histogram represents all ~9000 species-season-cell-year (SSCY) combinations with ≥30 observations. (A) Changes in activity onset time (10th percentile of daily observations), where negative values indicate earlier onset after correction. (B) Changes in activity offset time (90th percentile of daily observations), where positive values indicate later offset after correction. (C) Absolute changes in activity duration (difference between 90th and 10th percentiles), where positive values indicate longer activity periods after correction. (D) Percent changes in activity duration relative to uncorrected estimates. Black dashed lines indicate no change (zero); colored solid lines show mean values for each metric. Bias correction shifted activity onsets earlier by an average of 0.43 hours, extended offsets later by 1.03 hours, and increased estimated activity duration by 1.46 hours.


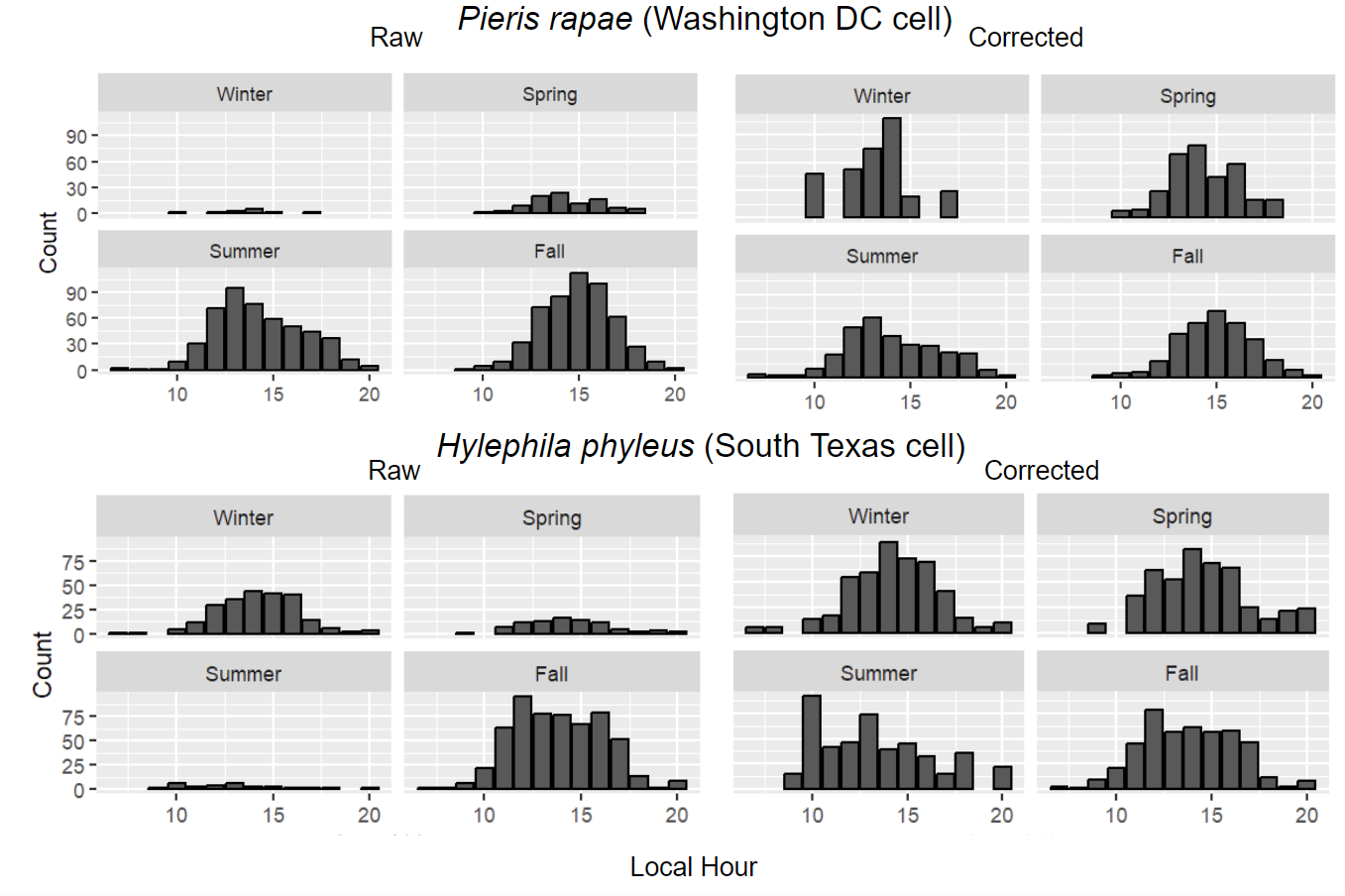


**SI Figure 3. Results of seasonal adjustment of activity curves of selected species-cells.** Raw versus bias-corrected seasonal activity curves are shown for two selected butterfly species and cells. Each panel contains the observations from one species-season-cell, with the left panels showing raw curves and the right corrected curves, collectively showing how our bias correction with weighted bootstrapping corrects for poorly sampled seasons and times within seasons.


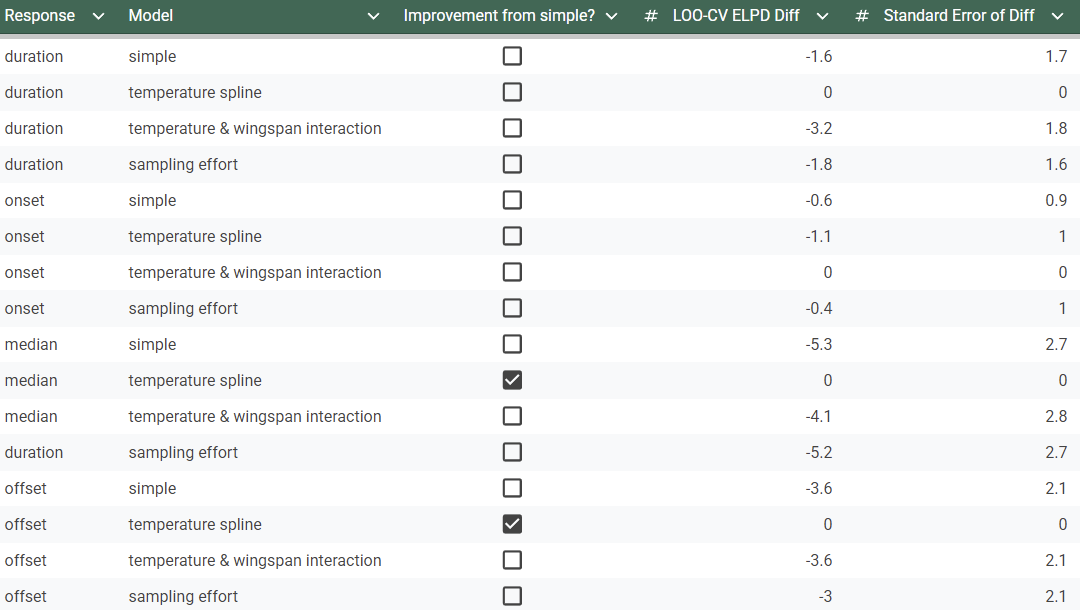


**SI Table 1. Model comparison for different response variables** using Leave-One-Out Cross-Validation Expected Log Predictive Density (LOO-CV ELPD). The table presents model fits for four response variables: duration, onset, median, and offset. Each response variable is modeled using three approaches: (1) a simple model, (2) a model incorporating a temperature spline, and (3) a model with a temperature and wingspan interaction. The LOO-CV ELPD difference is reported relative to the best-performing model for each response variable, with lower values indicating worse model fit. The standard error of the difference is also reported. Models that provide an improvement over the simple model are indicated with a checkmark.


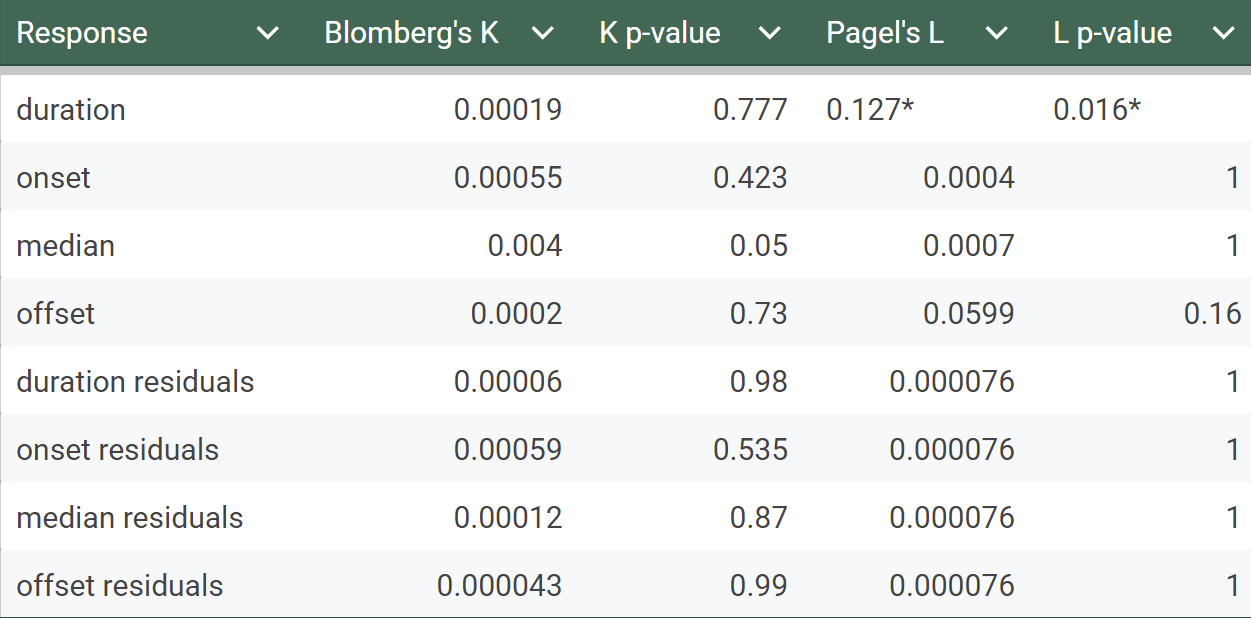


**SI Table 2. Assessment of phylogenetic signal of activity metrics using Blomberg’s K and Pagel’s λ**, calculated using the kTest and lambdaTest functions in the phylosignal package, respectively. Shown are K values, associated p-values, λ values, and p-values testing the null hypothesis that λ = 0. Phylogenetic signal was tested for raw activity metrics (duration, onset, median, and offset) as well as the residuals of the top models for each metric, allowing for an assessment of phylogenetic conservatism after accounting for model covariates. Significant results (p < 0.05) are marked with an asterisk (*).


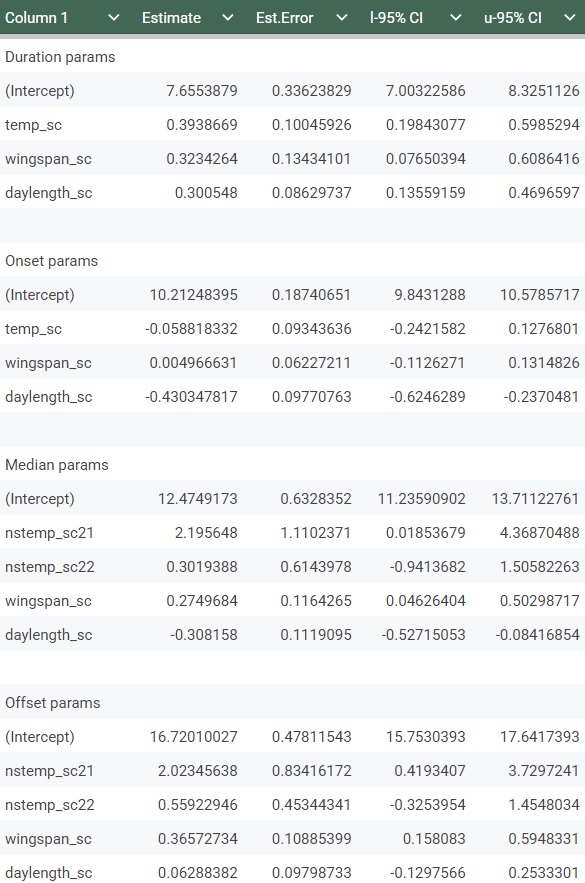


**SI Table 3. Results of phylogenetic generalized linear mixed models (PGLMMs)** fit for the best models for onset, median, offset (termination), and duration of daily activity patterns after model selection. The models were fit in a Bayesian framework using *brms* with default flat priors, and convergence was confirmed (Rhat < 1.01, effective sample size > 400, no divergent transitions). Model parameters are presented as posterior means with standard errors, along with the 95% credible intervals.
