# Supplementary material for: Using citizen science data to estimate trait and climate drivers of daily activity patterns in temperate butterflies
Source: PLoS One. 2025 Nov 21;20(11):e0335856. doi: 10.1371/journal.pone.0335856 (PMC12637895; doi:10.1371/journal.pone.0335856)
Supplement: Attachment — Submitted filename: Butterfly Activity Reviews & Responses.docx [file pone.0335856.s004.docx]

Response to reviewers for “Using citizen science data to estimate trait and climate drivers of daily activity patterns in temperate butterflies”

**Editor Point-by-Point**

“We require you to either (1) present written permission from the copyright holder to publish these figures specifically under the CC BY 4.0 license, or (2) remove the figures from your submission”

We have amended the figures to include only images with a license compatible with CC BY 4.0, and specified the sources of the images in the figure legend as follows. “Photo 1 by Thomas Shahan, CC BY 2.0 (https://flic.kr/p/2jBjNxc). Photo 2: Judy Gallagher, CC BY 2.0 (<https://flic.kr/p/2qaRGQj>). Photo 3: Thomas Shahan CC BY 2.0 (<https://flic.kr/p/2qn1WKb>). Photo 4: John Villella, Public Domain (<https://flic.kr/p/umwr7p>). Photo 5: Matt Pelan CC BY 4.0 (<https://www.inaturalist.org/observations/271745496>). Photo 6: Judy Gallagher, CC BY 2.0 (<https://flic.kr/p/2kSrpbt>). Photo 7: Hill Craddock, CC BY 4.0 (<https://www.inaturalist.org/observations/298955795>).” There was not a specific format for image attribution on the PLOS page ([https://journals.plos.org/plosone/s/licenses-and-copyright#loc-give-proper-attribution](https://journals.plos.org/plosone/s/licenses-and-copyright#loc-give-proper-attribution)) so please let me know if the format should be changed. We apologize for our oversight with Fig. 1 and have added attribution there as well in the revised manuscript.

“Please include captions for your Supporting Information files at the end of your manuscript, and update any in-text citations to match accordingly. Please see our Supporting Information guidelines for more information: <http://journals.plos.org/plosone/s/supporting-information>.”

We have added this information to the manuscript file.

**Reviewer 1 Point-by-Point**

“I missed some statement about the accuracy of species identifications. This is a notorious challenge with citizen scientists, and I doubt a bit whether the iNaturalist procedure (two persons concur in their assessment, and this already defines a ‘research grade’ data point) is really that convincing, when it comes to clades with many similar looking species
For example, in Fig 3 there are various Erynnis species noted, which look quite similar to another. How robust and reliable are species IDs in these cases? Other genera where multiple similar species occur, include Phyciodes, Chlosyne, and some more. I am not sufficiently familiar with the US butterfly fauna to provide more detailed suggestions at that point.
 I was surprised to see only one Celastrina species (viz. C. ladon). The taxonomy of the genus Celastrina in North America is surely complicated, see for example. https://doi.org/10.3897/zookeys.584.7882 and multiple references therein. This raises the question whether all observations noted as ‘ladon’ in GBIF are truly referable to this particular species? This distinction could be relevant for your analyses, since the thermal environments of activity differ between the spring and (late) summer species in this complex taxon of blue butterflies… At any rate, the topic of identification accuracy and reliability should be addressed in the discussion, since this is generally a serious issue with citizen science data on insects.”

We agree with the sentiment and that this issue should be acknowledged, but we also note that most identifications are made by taxonomic experts, many of whom are trained classically in taxonomy (Campbell et al. 2023; White et al. 2023). For example, the top identifier of *Celastrina ladon* is Dr. Nick Block whose research focuses in part on the speciation and hybridization of butterflies in the United States. This expertise of identifiers means that the bulk of the identifications occurring on iNaturalist are done by professionals who will not identify photos of organisms beyond the taxonomic resolution that a photo allows. In the case of *C. ladon*, most are not research grade and only those that are research grade are used for this analysis. Still, some inaccurate ids are expected, but we expect these inaccuracies to add noise rather than systematic bias given our large sample size and macro-scale perspective. Further, we expect that most misidentifications are between closely related species, and the use of phylogenetic autocorrelative approaches in model fitting also helps mitigate this issue. We treat species not as fully independent units, but account for relatedness. Summarizing your valid concerns and our thoughts, we added the following sentence to Methods: “We acknowledge that species identifications in iNaturalist are not perfect and errors exist, but note that the majority of identifications are made by taxonomic experts (44, 45) and we expect occasional inaccuracies to add noise, rather than systematic bias, given our large sample size and macro-scale perspective.”

“Overall, I missed a list of species included in the analyses, plus a reference to what taxonomy you are adopting (to use the Celastrina example again: depending on the taxonomy adopted, there are somewhere between 3-9 species in North America).”

We have added a list of species included in the study to the Supplemental Information. As our iNaturalist data comes from GBIF, we use the GBIF Backbone Taxonomy (<https://www.gbif.org/dataset/d7dddbf4-2cf0-4f39-9b2a-bb099caae36c>), and have added text referencing this.

“Another aspect I missed when reading your paper was the potential influence of butterfly sex on flight activity. It is well known that, for example, males of many butterfly species switch between patrolling and perching for locating females. This shift is related to temperature (and thus time of day), with perching prevailing under cooler (morning) conditions, while patrolling becomes more common at warmer (noon) temperatures. Also, in some species females are predominately flying in forest canopies, whereas males also seen at ground level. Hence, likelihood of observing a butterfly may be contingent upon its sex. I admit it would be impossible to check all these >1 million records for sex of the observed specimens, but this theme should clearly be touched upon in the discussion.”

Thank you for this insightful comment. We feel that such an analysis is beyond the scope of our project, especially given the lack of sex annotations in iNaturalist as you mention—but would be interesting to examine in follow-up work. We have added the following sentences in the Discussion noting the potential importance of sex: “Another relevant factor is butterfly sex, as the microhabitat use differs across sexes in many species, including canopy preference, thermoregulatory needs, and time-of-day activity. In highly dimorphic species one sex alone could be driving some of the patterns we observe, especially if it has higher detectability than the other.”

“L 71: in the Lepidoptera, some species are even cathemeral (though this does not apply to true butterflies in temperate zones):
 https://doi.org/10.1111/brv.13024
 https://doi.org/10.1098/rsos.250543
 <https://doi.org/10.1038/s41467-024-45329-5>”

We have added this note to line 71.

“ L 78 and occasionally throughout the paper: take care of a consistent mode of citing references. PLOS demands a numeric system, so please adhere to that everywhere.”

We have amended citations to be numeric.

“ L 139: would one really expect that small-sized butterflies are less efficient in convective cooling? I would rather state the opposite is true: smaller bodies lose heat more quickly, based on the ratio of thorax volume (this is where heat is generated) and body surface (where heat is lost). See for example here, for some US butterflies of different sizes occurring in cool environments:
 <https://doi.org/10.1086/physzool.59.6.30158609>”

We appreciate this comment and agree that, from a biophysical perspective, smaller butterflies have a higher surface-area-to-volume ratio and thus a greater potential for passive convective heat loss. We have added a reference where field data indicated that smaller butterfly species’ body temperatures track ambient air temperature much more closely than those of larger species, which exhibit significantly greater thermoregulatory buffering (Bladon et al., 2020). Thus, despite their theoretical cooling advantage, we believe that our expectation that small butterflies are more vulnerable to rapid heat gain and overheating under natural conditions holds up to scrutiny, and have revised the corresponding intro paragraph as follows to clarify:

“We also predict that smaller butterflies will be more sensitive to daily and seasonal temperature changes. Although from a biophysical perspective smaller butterflies have a higher surface-area-to-volume ratio and thus a greater potential for passive convective heat loss, field data has indicated that smaller butterfly species’ body temperatures track ambient air temperature much more closely than those of larger species, which exhibit significantly greater thermoregulatory buffering (48). Because of the generally weaker buffering ability of smaller butterflies, we predict that they will be less active under low temperature conditions and during periods away from the thermal peak of the day. Likewise we predict that excessive heat (hot temperatures and the daily thermal peak) will also pose a greater challenge for smaller butterflies and result in reduced activity. In addition, we predict a more gradual “warm-up” period for larger butterflies that could result in increased activity later in the day. Finally we predict that diel activity timing might be phylogenetically conserved, especially if thermal niche and its relation to body size is also conserved.”

“ L 157: excluding crepuscular records is certainly valid for the US fauna, but in tropical regions truly crepuscular activity does occur in certain Papilionoidea species. Hence, if your approach is transferred / generalized to other biomes, this procedure needs to be adapted.”

We agree and have added the following sentence in the discussion bringing up this point: “While we expect that excluding records before 8 AM or after 8 PM as error is valid for USA butterfly fauna, in tropical regions the presence of crepuscular taxa warrants a more holistic approach.”

“ Figure 1: at least in my review file the pie chart diagrams were distorted to elliptical shape. Care should be taken to provide a non-distorted version for publication.”

Fixed in the revised manuscript.

“ Figure 2: the X-axes in this graph require attention. I did not understand why there are different scalings, and what the isolated ‘axis’ in the middle of the graph might represent.”

Since the magnitude of the temperature effect on median and offset is much greater than the other effects, keeping that axis scale across all effects makes the other effects difficult to see and interpret. We agree that an explanation in the legend is necessary and added the following: “Due to the increased magnitude and credible interval size of the effects of temperature on offset and median, we present those distributions on a different x-axis scale (dashed line) as to not visually obscure the other effects.”

“ Figure 3: the cladogram has very poor resolution. Please provide a graph in high resolution, and where the scientific names of the organisms are really in line.”

Improved in the revised manuscript.

“In the methods section you state that you have deleted Danaus plexippus from your analyses, but in this graph the monarch is still included. What now is correct? Inclusion or exclusion?”

This was an error on our end to include Danaus plexippus in the figure - we have removed it.

“In line 284 you state your analyses cover 174 species, but the cladogram does NOT show them all. It is really crucial (in the sense of open science) that a complete list of those species which entered into your analyses (i.e. which you retained after filtering the raw data) is reproduced in the paper.”

The cladogram shows a sample of the species used as a cladogram with all 174 species would lack visual clarity. We chose which species to include based on which are best sampled and thus have the best species averages for diel activity metrics, with the sampling cutoff being n=15, and have now mentioned this in the figure legend. As mentioned in a previous response, we also added a list of species in the analysis to the SI.

**Point-by-Point Reviewer 2 Comments**

“The methods are described well and the results are mostly justified. However, there are a few statistical issues relating to the asserted similarity between distributions, effects of hourly rounding on the bias adjustment, and potential overfitting, that need to be resolved before I can recommend it for publication. These and a few other (mostly very minor) revisions are described in detail below.

“The GitHub repository for data processing and analysis linked in the submission table (https://github.com/jidec/inat-butterfly-activity-analysis) is not available.”

We apologize for not making this available sooner and doing so was an oversight - the repo has been made public at the same link (<https://github.com/jidec/inat-butterfly-activity-analysis>).

“ L158–160: Does this correspond to the typical seasonality of butterflies in this study area? Or were these seasons primarily chosen to represent human activity?”

This is a good question - the four seasons were primarily chosen to represent human activity, because the purpose of this aggregation was bias reduction. We have added the following sentence for clarification: “We elected to use the four seasons as the temporal dimension of bias because it is a straightforward and interpretable factor that is generally in line with the yearly cycles of both climate and human observer behavior.”

“L184: “grids cells” → “grid cells”?
 L195: “old” → “existing” to avoid ambiguity with personal age?
 L201: “GAM” (presumably Generalised Additive Model) is not defined in the text.
 L205: Mixed present/past tense again.”

L227: The 90% value is called “termination” here but was called “offset” earlier. It would be good to use one word consistently.

L246: “VIF” is not defined in the text.
 L247: “problemenatic” → “problematic”?
 L249–252: [1] and [2] vs. 1) and 2) earlier in the text.

L267: “Pagels” → “Pagel’s”.”

L283: Inconsistent use of thousands separators (comma earlier, none here).
 L304: Should Figure 3 come before Figure 2?
 L304: Double . at the end of this sentence.
 L311–312: Mixed tense again.
 L367: “Efforts … is” is grammatically incorrect.

Response / notes:

All fixed in the revised manuscript.

L300: Should this reference SI Table 2 rather than 1?

Referencing SI Table 1 should be correct here: “The top model for median and offset included the spline fit for temperature, while the top model for duration and onset was the basic model (SI Table 1).” as SI Table 1 includes the model selection information.

” L205–207: Why is this weighting applied?”

The weighting is the step when the bias reduction is actually applied to the data - we have amended that sentence to clarify this: “The bias reduction is applied when we use the inverse sampling effort as weights during bootstrapping, upweighting observations that go against the grain of the bias and downweighting those most associated with it.” See also our response below where we added a figure helping to clarify bias reduction.

” L213: Am I right to assume you used daily temperature data rather than average climatologies?”

Because we used SSCY combos, we used the average climate of each season-cell-year.

” L241–244 & SI Figure 1: Visually, I’m not sure I agree with the assertion that the distributions within each panel are similar and / or broadly gaussian. Duration has a clear skew; Offset appears bimodal; Onset and Median look almost like offset sawtooth curves. The peaks due to the hourly rounding appear quite large, certainly too large to neglect out-of-hand as is done in the text. Please elaborate further on this issue, e.g. by quantifying the similarities between the distributions, calculating the effects of the hourly peaks on the bias adjustment process (e.g. compared to half-hourly bins or rolling averages)**,** etc.”

We agree that our wording could be improved in this section of the manuscript. We believe our models actually do a good job of predicting more realistic diurnality than our observed data that has the bias-reduction caused peaks. We have modified our sentence to read, “Peaks in the observed data are caused by time of observation rounded to hours during the bias-reduction process. However, our models fit assuming a gaussian process broadly conform to the observed empirical data while smoothing out the artifactual peaks.”

“…are the colours in the figure consistent between panels, legend, and caption?”

Yes - in Fig. 1 the colors in the pies match those in the legend. We use different colors on the right-hand panel between they correspond to diel activity rather than season.

”L256–261: (How) does this approach account for overfitting, since you are comparing models with different degrees of freedom?”

This approach is relatively robust to overfitting because expected log predictive density (ELPD) for new data via leave-one-out cross-validation (LOO-CV) examines prediction accuracy of out-of-sample values, and does not measure a goodness of fit. Thus, an overfit model would have low predicted accuracy of out-of-sample values and would not be chosen as the top model.

”L258: Leave-one-out cross-validation has some limitations in its ability to really capture out-of-distribution samples (see e.g. doi:10.1016/j.rse.2025.114820). Do you expect this to affect the results here, or is the issue not relevant?”

We appreciate this statistical point from Werther et al. and acknowledge that predictions should be interpreted cautiously when extrapolating beyond the spatial, phylogenetic, and climatic scope of our study. In our case, it is helpful that our aggregation produces a largely consistent gradient and our mixed-effects structure explicitly models the variation sources (location, phylogeny) that could create out-of-distribution scenarios. Future work could use spatial or phylogenetic block cross-validation as suggested by Werther et al. to more rigorously test extrapolation capacity, though this would require larger sample sizes per block.

“Before the bias-adjusted dataset is presented, I would like to see some results relating to the bias adjustment method itself. How large were the biases you found in the iNaturalist dataset? How much of the biases did your method remove? How does an SSCY curve change between original and bias-adjusted? This information would be highly valuable to any readers who want to replicate your study or apply the methodology elsewhere. This could be described at the start of the Results or in the relevant subsection of the Methods.”

We agree that this is a valuable addition to the manuscript that would better illuminate the bias-adjustment approach. We have added the following paragraph to the Results respectively along with two associated SI figures.

“*Quantifying the bias correction*

Our bias correction approach resulted in meaningful shifts across all activity metrics. The correction shifted activity onsets earlier by an average of 0.43 hours and extended activity offsets later by an average of 1.03 hours. These changes combined to increase estimated activity duration by 1.46 hours on average, representing a 27.3% increase relative to uncorrected estimates. Duration showed the largest change as observations in the morning and evening, times with little observer effort, are most strongly upweighted. These corrections help to address the systematic oversampling during the summer and afternoon hours and undersampling during the winter and morning hours in community science observations. When visualizing the seasonal corrections made to the curves, the bias adjustment had the most pronounced effects on winter activity patterns, where low observer effort during cold morning hours resulted in substantial upweighting of rare early observations. Conversely, summer patterns showed more modest corrections as observer effort was more evenly distributed throughout daylight hours, though afternoon peaks were still reduced through downweighting of oversampled time periods.

SI Figure 2. Impact of observer bias correction on butterfly daily activity metrics. Distribution of changes in activity timing and duration after applying bias correction to account for temporal sampling biases in iNaturalist observations. Each histogram represents all ~9000 species-season-cell-year (SSCY) combinations with ≥30 observations. (A) Changes in activity onset time (10th percentile of daily observations), where negative values indicate earlier onset after correction. (B) Changes in activity offset time (90th percentile of daily observations), where positive values indicate later offset after correction. (C) Absolute changes in activity duration (difference between 90th and 10th percentiles), where positive values indicate longer activity periods after correction. (D) Percent changes in activity duration relative to uncorrected estimates. Black dashed lines indicate no change (zero); colored solid lines show mean values for each metric. Bias correction shifted activity onsets earlier by an average of 0.43 hours, extended offsets later by 1.03 hours, and increased estimated activity duration by 1.46 hours.

SI Figure 3. Results of seasonal adjustment of activity curves of selected species-cells. Raw versus bias-corrected seasonal activity curves are shown for two selected butterfly species and cells. Each panel contains the observations from one species-season-cell, with the left panels showing raw curves and the right corrected curves, collectively showing how our bias correction with weighted bootstrapping corrects for poorly sampled seasons and times within seasons.

“Figure 1: The colours used for Fall and Summer are difficult to distinguish for individuals with red-green colour blindness. Personally, I can see the difference clearly in the legend but they blend together in the pie charts. I would recommend changing (one of) the colours based on a standard colour scheme (e.g. https://colorbrewer2.org/#type=qualitative&scheme=Paired&n=4) and/or testing using a simulation tool (e.g. <https://www.color-blindness.com/coblis-color-blindness-simulator/>).”

Thanks for this comment – we are also committed to making our visualizations accessible. We selected our color palette using a colorblind-friendly palette selector, and double-checked it using a red-green colorblind (protanopia) simulation screen overlay, “Sim Daltonism”.

“L291–L296 & SI Table 1: See previous comment on overfitting. Why is temperature spline an improvement for median but not for duration, when both have LOO-CV ELPD Diffs of 0, indicating they were the best fits? This table might be clearer if the third column marked the best-performing model and the differences were compared to the simple model rather than to the best-performing (which is not always the same) – or if the absolute LOO-CV ELPD values were provided. For offset, are the differences in LOO-CV ELPD used for picking the temperature spline model significant considering the standard errors?”

Thank you for identifying these errors - upon rerunning the model comparison some numbers were found to have been placed in the table in error, and the new SI Table 1 has fixed this. However, the correct numbers still have only the temperature spline fit significantly winning out over the simple for median and offset, as is described in the manuscript.

“L292–293 & L295–296: “No models were improved when including the temperature-wingspan interaction or the sampling effort term” vs. “Number of observations did not improve any model, so we removed it as a predictor.” – are these sentences redundant compared to one another?Furthermore, for replicability, please quantify these results rather than simply asserting them.”

You are correct that the second sentence is redundant and we have removed it. We also add the model selection info for the number of observations to SI Table 1 as requested.

“ L412–419: This section states that there was no significant shift in onset for small butterflies, and then says this is consistent with the literature which states that larger butterflies have activity patterns shifted later into the day and that smaller butterflies prefer the early morning. Aren’t those statements inconsistent? Or am I misunderstanding?”

We understand the confusion - here we are referring to the scenario where offset, rather than onset, shapes early vs. late activity. While both small and large butterflies start around the same time, large butterflies end much later, showcasing that smaller butterflies prefer the early morning. We have amended the text to clarify.
